# Challenges and successes for the grantees and the Technical Advisory Group of WHO’s influenza vaccine technology transfer initiative

**DOI:** 10.1016/j.vaccine.2016.07.047

**Published:** 2016-10-26

**Authors:** Gary Grohmann, Donald P. Francis, Jaspal Sokhey, James Robertson

**Affiliations:** a Environmental Pathogens, Canberra, ACT, Australia; b Global Solutions for Infectious Diseases, South San Francisco, CA, United States; c Consultant in Vaccinology, India; d Consultant in Virology, UK

**Keywords:** Influenza, Vaccine manufacture, Less developed countries, Technology transfer

## Abstract

One of the aims of the WHO Global Action Plan for Influenza Vaccines (GAP) was to transfer influenza vaccine production technology to interested manufacturers and governments in developing countries, to enable greater influenza vaccine manufacturing capacity against any pandemic threat or pandemic. For this objective, the GAP was supported by an independent Technical Advisory Group (TAG) to assist WHO to select vaccine manufacturing proposals for funding and to provide programmatic support for successful grantees. While there were many challenges, for both the TAG and grantees, there were also notable successes with an additional capacity of 338–600 million pandemic vaccine doses being made possible by the programme between 2007 and 2015, and a potential capacity of more than 600 million by 2016/17 with up to one billion doses expected by 2018/19. Seasonal vaccine production was also developed in 4 countries with another 4–5 countries expected to be producing seasonal vaccine by 2018/19. The relatively small WHO investments – in time and funding – made in these companies to develop their own influenza vaccine production facilities have had quite dramatic results.

## 1 Introduction

Influenza viruses provide a constant disease threat to humanity in the form of regular seasonal epidemics, novel pandemic threats and pandemics of varying severity. The burden of disease varies from region to region and year to year, and the health and economic consequences of this disease are profound. To be prepared for these occurrences requires global cooperation and the interaction of many private and public stakeholders including manufacturers, researchers, health providers, policy makers, expert committees and specialist laboratories, as well as the guidance of health authorities, regulators and international organisations such the World Health Organization (WHO). WHO provides a number of essential programmes, policies, and committees related to influenza such as the International Health Regulations (2005) (IHR), including IHR emergency committees; the WHO Strategic Advisory Group of Experts on Immunization (SAGE); and more recently the Pandemic Influenza Preparedness (PIP) Framework, which focuses on equity in virus sharing and benefit sharing [1]. Importantly, there is also WHO’s Global Influenza Surveillance and Response System (GISRS), which brings together over 140 laboratories in a world-wide influenza virus surveillance network responsible for virus characterisation, risk assessment, virus sharing, and ultimately the selection and production of candidate vaccine viruses. This network is essential for alerting the world of any emerging influenza threats and for the eventual provision of seasonal and pandemic vaccines by manufacturers [2]. However, despite this global effort, it has been recognised for some time that in the event of a pandemic there would be a significant shortfall of available pandemic vaccine as demand would be far greater than supply. The re-emergence of H5N1 in Southeast Asia in 2005 focused the world’s attention on these issues, with developing countries calling for greater vaccine production capacity in their own regions and for more equitable benefit sharing. Moreover, it was also clear that low- and middle-income countries (LMIC) would be the most affected by any pandemic influenza threat. Therefore, in 2006, the WHO published a Global Action Plan for Influenza Vaccines (GAP), which is a comprehensive strategy to reduce the present global shortage of influenza vaccine. One of its objectives involved the transferring of influenza vaccine production technology to interested manufacturers and governments in LMICs, to enable local and overall increased influenza vaccine manufacturing capacity when a pandemic threat or actual pandemic occurs [3].

## 2 The Technical Advisory Group and method of work

In 2007, an independent Technical Advisory Group (TAG) was formed to assist WHO in selecting vaccine manufacturers’ proposals for funding under the GAP. Each TAG member brought with them considerable expertise in a variety of areas including influenza vaccine development, vaccine production, biotechnology, and/or relevant regulatory experience, which allowed the committee to understand the challenges ahead for both the grantees and the WHO and to give appropriate advice. Table 1 lists current and former members of the TAG.

In the initial phases, developing country vaccine manufacturers were invited to submit letters of intent, via a public invitation on the WHO website, declaring their interest in developing influenza pandemic vaccine capacity. These letters were reviewed against several criteria including public health value, potential regional impact, technical merit, the level of government support and probability of success. As described previously [4], eligible manufacturers were then invited to submit full proposals, which were scored, ranked and weighted by TAG members according to an evaluation of five elements: the project plan, the staffing and management plan, performance measures, an understanding of the requirements, and the budget justification. An ongoing programmatic review was also instituted for the successful grantees involving regular site audits, a review of government (and other) support, sustainability, an assessment of Good Manufacturing Practices (GMP) and biosafety requirements, a review of the results of research and method development, a review of data on vaccine production and clinical trials, and an assessment of progress towards registration of a pandemic vaccine. TAG members also reviewed the quarterly reports submitted to WHO by the grantees and held regular teleconferences in addition to the annual face-to-face TAG meetings. TAG meetings were often held at one of the grantee sites to provide an opportunity for direct consultation. Over the life of the programme 14 manufacturers were given financial support over four review processes in 2007, 2009, 2011, and 2013 (Table 2). The Institute of Experimental Medicine (IEM), St. Petersburg, Russian Federation was also given financial support for research and development of live attenuated influenza vaccines (LAIV) candidates and the reconstruction of its laboratory. In total, approximately US$50 million was allocated by the WHO to grantees.

## 3 Challenges

Most of the countries involved had never manufactured influenza vaccine and did not have an established influenza vaccine delivery programme. Therefore proposals had to be developed for building production facilities as well as for eventual vaccine delivery. It became clear from a manufacturing and quality perspective that the development of seasonal vaccines would logically precede the development of pandemic vaccine. This would ensure that all the manufacturing processes and associated quality and consistency issues would be in place, so that pandemic vaccine could be reliably produced when needed. Moreover, from a vaccine delivery perspective, government policies and appropriate infrastructure needed to be in place as part of pandemic preparedness. Manufacturers were encouraged to communicate frequently with regulatory authorities and relevant health and other government ministries to ensure that such programmes were in place. Moreover, grantees were encouraged to consider and develop local and regional markets and obtain contracts for seasonal and pandemic vaccine production.

The complexities of influenza vaccine manufacture are unique and can be fickle. On WHO’s advice, grantees generally chose to invest in egg-based production methods, and some later worked with cell culture-based systems as well. Many wanted to begin with pandemic vaccine production because of the H5 threat but all were encouraged to establish pilot programmes and begin with one or more seasonal vaccine viruses. As few of the grantees had previous experience of influenza vaccine development and manufacture, they all required training and, for this purpose, WHO established a centre of excellence and training at the Netherlands Vaccine Institute (NVI) in Bilthoven. NVI established a pilot-scale production process that could be transferred to manufacturers [5]. Specific training modules were also provided by the following institutions: the National Institute for Biological Standards and Control (NIBSC) in the United Kingdom, the adjuvant technology transfer hub at the University of Lausanne, Switzerland [6], the Utah State University Center for Integrated Biosystems, and the North Carolina State University Biomanufacturing Training and Education Center (BTEC) in the USA.

To support egg-based production, most recipients had to build both their own egg supply facilities and their own production factories. While many were successful in this venture, two companies encountered unforeseen issues which either delayed or halted the programme. Three grantees started with fill/finish facilities using bulk vaccines supplied by other established manufacturers with mixed success. Several grantees had issues with the splitting and inactivating processes, procurement of equipment, quality control and/or quality systems. Some companies lacked the financial resources to continue smoothly with influenza production which caused delays. Border control was also an issue for most grantees as viruses and candidate vaccine viruses had to be correctly acquired and handled in appropriate BSL2/3 conditions.

Apart from issues with seasonal vaccine production and security, there were also challenges to producing clinical lots and clinical protocols for pandemic vaccines. GMP was sometimes an issue and the decisions around making split or whole virus vaccines, with or without adjuvant, for clinical trials, as well as dose regimes, required guidance from the TAG and local authorities. Several grantees were successful in undertaking a number of clinical trials but others struggled or failed to meet regulatory criteria. The TAG had to bring in other consultants to help solve some specific problems and also provide continuing advice to grantees on these issues.

## 4 Successes

Feedback from the grantees indicated that the training courses provided were instrumental for the successful implementation of the projects. The grantees also highlighted the benefit of having WHO involved, both via finance and expertise. Such involvement provided intangible support, giving confidence to governments and other funders of the high standard of the projects. Moreover, independent external WHO reviews of the projects helped assure companies and governments that their investments were reasonably managed and had a high probability of technical success. These reviews also gave confidence to the local employees and researchers working on the projects to continue to move forward with their aim of producing influenza vaccines. In addition, it is important to highlight that in nearly every case the WHO grants were small in relation to the overall investment that companies and/or governments made. The overall WHO investment of only US$50 million leveraged a total of just under US$1 billion in funding by governments or other bodies. The vast majority of these funds were invested in infrastructure by the grantees.

The overall success of the programme, in terms of vaccine production, is summarised in Table 3, which lists the successful grantees and the products produced over time as well as the projected pandemic capacity of about 1 billion doses expected to be available by 2019, pending the results of clinical trials in some cases. These numbers are based on actual and expected seasonal vaccine production and were derived from manufacturers themselves and the opinions of the TAG members. The subsequent projections of monovalent pandemic vaccine capacity is an estimate based on three times the seasonal production capacity. If a billion doses can be contributed at a time of crisis, then these grantees would have contributed enormously to regional pandemic preparedness. Moreover, H1N1 vaccines were also produced by several manufacturers and were approved in India, Korea, Romania, Thailand, whereas H7N9 clinical lots were developed by manufacturers in Brazil, China, India, Indonesia and Viet Nam. Nearly all manufacturers produced small amounts of H5N1 vaccine.

Some grantees chose to develop influenza production facilities in partnership or collaborate with large international pharmaceutical companies (Instituto Butantan, Brazil and Birmex, Mexico with Sanofi Pasteur; The Government Pharmaceutical Organization (GPO), Thailand with Kaketsuken; Biovac, South Africa with bioCSL; and Bio Farma, Indonesia with Biken). Independent of WHO, these recipients have made their own business arrangements with their technology transfer partner either to construct production facilities or to share technologies involved in the production, fill finish or other components of the larger production process to produce influenza vaccines. Furthermore, the Serum Institute of India (SII), GPO, and Changchun BCHT Biotechnology Co. (BCHT) successfully collaborated with IEM to begin the production of LAIVs.

## 5 Conclusions

The ultimate goal of this initiative was to increase access of safe, effective and affordable pandemic influenza vaccines to developing country populations. The successes have been impressive with an expanded world capacity of an additional 338–600 million pandemic vaccine doses being made available between 2007 and 2015, and a potential capacity of more than 600 million by 2016/17 and up to one billion doses by 2018/19. Moreover, seasonal vaccine production has already been developed in 4 countries with another 4–5 countries expected to be producing seasonal vaccine by 2018/19. Pandemic vaccine has also been developed in some countries, notably against H1N1, H5N1 and H7N9. Challenges remain for the successful grantees to keep moving forward with their programmes and also for those still developing product to continue to do so. To ensure ongoing success in the future, some degree of technical support and training is still needed for all the grantees and financial support for those hoping to produce vaccine in the future.

Much has happened in the last ten years with regard to pandemic influenza preparedness while WHO’s GAP was in progress. WHO revised the IHR as well as established the PIP Framework, which provides equity in virus and benefit sharing. There have also been important meetings under the WHO GISRS umbrella [7], notably consultations on improving influenza vaccine virus selection [8] and addressing the issues around switching from seasonal to pandemic production during the start of a pandemic [9]. Also the Nagoya Protocol, which provides equity in access and benefit sharing to genetic resources, entered into force in 2014 [10]. Soon after the establishment of the GAP a mild pandemic occurred in 2009 and there have subsequently been several pandemic threats - notably the emergence of H7N9, the re-emergence of H5N1, and late emerging H1N1 and H3N2 genetic subgroups causing more severe disease than expected.

There have also been technological advancements, with quadrivalent seasonal vaccines, high dose seasonal vaccines, and new cell culture vaccines coming onto the market as well as an influenza vaccine made using recombinant technologies, which was successfully licensed in the USA [11]. Moreover, there have been advances in dose sparing strategies including the development of adjuvants and novel delivery systems, which are almost certainly necessary to ensure the availability of enough pandemic vaccine for the world [12], [13], [14]. The ability now to produce synthetic viruses from genetic data will improve virus sharing and the production and availability of candidate vaccine viruses. All these positive advances, as well as the continuing global research effort, will help the world to prepare for the next influenza pandemic threat. Through the GAP programme and other WHO programmes and resources, as well as the work of manufacturers and researchers, the world is better prepared, and developing country regions are now also better prepared, for any emerging influenza pandemic.

## Conflict of interest statement

The authors state they have no conflict of interest.

## Figures and Tables

**Table 1:** Current^∗^ and previous members of the TAG.

TAG member	Particular expertise	Membership
John Boslego	Vaccine production	From 2007–October 2014
Rick Bright^∗^	Biotechnology	From June 2011
Armen Donabedian	Biotechnology	From December 2008–June 2011
Donald Francis^∗^	Production of viral vaccines	From 2007
Gary Grohmann^∗^	Influenza vaccine and vaccine registration	From 2007
Michael Perdue	Influenza vaccine	From December 2008–April 2013
James Robertson^∗^	Influenza vaccine	From 2007
Jean-François Saluzzo	Production of viral vaccines	From 2007–November 2014
Jaspal Sokhey^∗^	Regulatory issues and Vaccine production	From 2007
Thomas Warf^∗^	Vaccine production	From September 2013
Gerd Zettlmeissl^∗^	Biotechnology and vaccine production	From June 2011

**Table 2:** List of WHO grantees by country, manufacturer, private/public status, and technology/product developed.

Country	Institute (Legal status)	Technology
*2007 grantees*		
Brazil	Instituto Butantan (Public)	Egg-based inactivated split and/or whole virion H5N1 with adjuvant
India	Serum Institute of India (Private)	Egg-based technologies: (i) whole virion alum-adjuvanted inactivated vaccine, and (ii) live attenuated influenza vaccine using WHO sublicensed Russian technology. Cell-based technologies: live attenuated using Russian technology
Indonesia	Bio Farma (Public)	Egg-based split vaccine. Cell-based technologies
Mexico	Birmex (Public)	Egg-based split vaccine. Fill-finish facility
Thailand	Government Pharmaceutical Organization (Public)	Egg-based technologies: (i) establishment of an egg-based split inactivated seasonal vaccine process, and (ii) live attenuated influenza vaccine using WHO sublicensed Russian technology
Viet Nam	Institute of Vaccines and Medical Biologicals (Public)	Egg-derived whole virion and alum adjuvanted H5N1 and H1N1 influenza vaccines. Split seasonal vaccine

*2009 grantees*		
Egypt	Vacsera (Public)	Egg-derived whole virion vaccine
Islamic Republic of Iran	Razi Institute (Public)	Egg-based vaccines
Republic of Korea	Green Cross Corporation (Private)	Alum adjuvanted whole virion H5N1 vaccine
Romania	Cantacuzino Institute (Public)	Pilot-scale production of seasonal egg-based inactivated split influenza vaccine
Serbia	Torlak (Public)	Egg-based inactivated vaccines

*2011 grantees*		
Kazakhstan	RIBSP (Public)	Egg based whole virion and split virion vaccines
South Africa	Biovac (Public/Private partnership)	A fill-finish facility for seasonal vaccine: split egg-based product

*2013 grantees*		
China	BCHT (Private)	Egg-based technologies: live attenuated influenza vaccine using WHO sublicensed Russian technology

**Table 3:** Successful grantees, product development status by time and pandemic vaccine production capacity.

						Pandemic production capacity⁎ in millions of doses			
						Actual		Predicted	
Country	Institute	Vaccine	Technology platform	By 2016/17	By 2018/19	2006	2015	2016/17	2018/19
Mexico	Birmex-Sanofi^a^	Seasonal	Inactivated, eggs	–	Approved	0	0	0	120^d^
Brazil	Butantan	Seasonal	Inactivated, eggs	Approved					
		Pandemic^b^		–	Mock dossier Approval	0	136	216	432
Romania	Cantacuzino	Seasonal	Inactivated, eggs	Paused					
		Pandemic			Mock dossier Approval	0	0	0	9
Serbia	Torlak	Seasonal	Inactivated, eggs	Phase III	Approved				
		Pandemic^b^		Animal studies	Phase II	0	0	0	3
India	SII	Seasonal	LAIV - eggs transitioning to cell culture	Approved	Cell Culture				
		Pandemic		Approved		0	20	200	>200
Thailand	GPO	Seasonal	Inactivated, eggs	Phase III	Approved				
		Pandemic	LAIV, eggs	Approved	Approved	0	1.5	1.5	30
Viet Nam	Institute of Vaccines and Medical Biologicals (IVAC)	Seasonal	Inactivated, eggs	Phase III	Approved				
		Pandemic^c^		Phase II	Approved	0	0	3	3
China	BCHT	Seasonal	LAIV - eggs	Phase II	Approval	0	0	0	150^d^
Republic of Korea	Green Cross Corporation	Seasonal	Inactivated, eggs transitioning to cell culture	Approved	Cell culture				
		Pandemic		Approved		0	180	180	>180
					**Total**	**0**	**338**	**600**	**>1127**

⁎ Calculated from either current or projected seasonal vaccine production. Numbers are approximate.

a Joint venture.

b Contains oil in water emulsion adjuvant.

c Contains alum.

d Based on seasonal production giving three times monovalent output of 15 μg pandemic vaccine.
